# Femtosecond Laser-Assisted Cataract Surgery: Analysis of Surgical Phases and Comparison with Standard Phacoemulsification in Uncomplicated Cataracts

**DOI:** 10.3390/vision6040072

**Published:** 2022-12-07

**Authors:** Antonio Baldascino, Matteo Mario Carlà, Federico Giannuzzi, Francesco Boselli, Tomaso Caporossi, Gloria Gambini, Antonio Villano, Aldo Caporossi, Stanislao Rizzo

**Affiliations:** 1 Ophthalmology Unit, Fondazione Policlinico Universitario A. Gemelli, IRCCS, 00168 Rome, Italy; 2 Ophthalmology Unit, Catholic University “Sacro Cuore”, 00168 Rome, Italy

**Keywords:** cataract, femtosecond laser assisted cataract surgery, cumulative dissipated energy

## Abstract

The aim of this work is to compare the time of surgical phases and the cumulative dissipated energy (CDE) of the phacoemulsification phase in femtosecond laser-assisted cataract surgery (FLACS) and in standard surgical procedures of phacoemulsification (PCS). This prospective, non-randomized study analyzed the data of 100 cataract surgeries, 66 using FLACS and 34 with standard PCS. The time of surgical phases was recorded by a digital chronometer; an additional parameter recorded was the CDE of the phacoemulsification phase. The mean time of femtosecond laser phase was 121.7 ± 27.3 s with minimal fluctuations in duration; the mean opening time of the corneal tunnel and the service incisions was 60.5 ± 20.4 s in the PCS, and 48.8 ± 17.4 s in FLACS (*p* = 0.04); the mean time of capsulorhexis was 39.6 ± 12.9 s in the PCS and 7.0 ± 5.2 s in FLACS (*p* < 0.0001); the mean time of phacoemulsification was 180.1 ± 45.6 s in the PCS and 163.0 ± 38.2 s in FLACS (*p* = 0.12); the mean aspiration time of the residual cortical was 66.3 ± 27.5 s in the PCS and 91.5 ± 35.7 s in FLACS (*p* = 0.02). Overall, the total surgical time of the cataract surgery was 742.3 ± 185.8 s in PCS and 985.1 ± 118.6 s in FLACS (*p* = 0.03). The mean CDE was 11.35 in the PCS and 8.3 in FLACS (*p* = 0.01). In conclusion, the greatest advantage obtained from the use of the femtosecond laser was the reduction of the duration of the phacoemulsification time and of the CDE parameter.

## 1. Introduction

Cataract surgery is the most commonly performed ophthalmic surgery in the world, with an estimated 19 million surgeries performed annually, representing approximately 83% of total ophthalmologic interventions. The World Health Organization estimates that the number of annual surgeries will increase, considering the incremental trend of the over-65 population, which doubled between 2000 and 2020. For this reason, the cataract surgery techniques are under constant review in order to improve efficiency, reduce costs, improve safety, and produce more reliable results [1].

During the last few years, the femtosecond laser (FL), already successfully used in keratorefractive surgery (LASIK) since 2001, Refs. [2,3] has begun to be used to make some of the surgical steps of cataract surgery: creation of corneal access; anterior capsulotomy; fragmentation of the nucleus of the crystalline lens; and creation of arcuate incisions for astigmatism [4,5,6].

Even though there is still much to know about the advantages and disadvantages of this technology in cataract surgery, it has been already demonstrated that the femtosecond laser allows for the planning and control of the size, shape, and centering of the capsulotomy, as well as the positioning and three-dimensional characteristics of the access incisions; it can also obtain a reduction of the ultrasonic power used during the phacoemulsification of the nucleus of the crystalline lens and simultaneously reduce the ultrasound emission time [4,7].

Crystalline lens density, surgical technique, and surgeon expertise affect ultrasound energy expenditure (CDE—cumulative dissipated energy) during phacoemulsification. Modern cataract surgery aims to reduce ultrasonic expenditure during cataract extraction to avoid collateral ocular tissue injury and postoperative problems related to higher CDE, such as corneal endothelial decompensation, corneal wound burn, and increased postoperative inflammation. In light of this, FLACS has been shown to be more effective in minimizing CDE, thus limiting post-operative complications [8].

The aim of this study is to compare the time of surgical phases and the cumulative dissipated energy (CDE) of the phacoemulsification phase in femtosecond cataract surgery (FLACS) and in the standard surgical procedure of phacoemulsification (PCS) in a real-world setting.

## 2. Materials and Methods

This is a prospective study that analyzed the data of 100 consecutive cataract surgeries performed between December 2020 and September 2021 by a single highly experienced surgeon (A.C.) in the same operating room of the Ophthalmology Department of the Gemelli Hospital in Rome with the use of a femtosecond laser [Alcon LenSx^®^ Laser System, Alcon, Fort Worth, TX, USA]. This research adhered to the tenets of the Declaration of Helsinki and was approved by the Catholic University of the Sacred Heart Ethical Committee in Rome, Italy. An informed and written consent was obtained from all enrolled patients.

Femtosecond laser-assisted cataract surgery (FLACS) was used in 66 cases, while in the remaining 34 cases, the operation was performed according to the standard surgical procedure of phacoemulsification with the micro-incisional technique (PCS). Case-by-case, the choice between FLACS and PCS was left to the surgeon. The phacoemulsification step was performed in both techniques using a last-generation phacoemulsifier [Alcon CENTURION^®^ Vision System, Alcon, Fort Worth, TX, USA].

According to the LOCS III classification system, [9] 57 cataracts were graded for density as 1+/2+ (38 in the FLACS group and 19 in the PCS group), and the remaining 31 as 3+/4+ (20 in the FLACS group and 11 in the PCS group), in order to compare surgical times between the two subgroups. The vital signs of all patients were monitored in the previous phases and during surgery, which was performed with topical anesthesia.

In each surgery (FLACS and PCS), the timings of the different surgical steps were registered by an expert ophthalmologist (A.V.) who assisted the surgery in the operating theatre, using a digital chronograph (OP Clock, Westerstrand) to assess the duration in seconds of all the steps of the procedure. The calibration of the chronograph was performed every day at the beginning of the operating room to ensure the reproducibility of the time points measured. The registered times were the following: (1) femtosecond laser phase time (exclusively in FLACS); (2) opening time of corneal wounds; (3) capsulorhexis/capsulotomy time; (4) phacoemulsification time; and (5) suction time of the residual masses. In each surgery, the total duration of the procedure was also measured from the docking in laser-assisted surgery and from the corneal incision by the blade in traditional surgery until the hydrosuture of the incisions, including all the times of instrument handling, microscope/patient centering, and intra-ocular lens (IOL) positioning into the cartridge. An additional parameter recorded was the CDE (cumulative dissipated energy) of the phacoemulsification phase. The data were put into an electronic database.

### Statistical Analysis

The statistical analysis was conducted using GraphPad PRISM Software (Version 9.0; GraphPad, La Jolla, CA, USA). Our sample’s normality was determined using the Shapiro-Wilk test, and a *p* > 0.05 was utilized to confirm the null hypothesis. We performed a *t*-test for unmatched pairs. To compare the difference between each pair of non-matched means, the Tukey test, which computes confidence intervals, was used. For contingency analysis, Chi-square and Fisher’s exact tests were utilized. In addition, correlation studies were performed on continuous variables. The quantitative results were represented as the mean standard deviation, and a *p*-value < 0.05 was deemed statistically significant.

## 3. Results

The population included 48 male patients (32 in the FLACS group and 16 in the PCS group) and 52 female patients (34 in the FLACS group and 18 in the PCS group), with a mean age of 69 years in the FLACS group (range, 42–84) and a mean age of 66 years in the PCS group (range, 26–93). The surgery was successfully completed without any intra- or post-operative complications in all cases but one, in which an anterior capsular rupture occurred during manual capsulorhexis with no vitreous loss and a single-piece soft IOL implant in the capsular bag. For this reason, this case has been excluded from our series. Moreover, one case of ineffective femtolaser capsulorhexis was reported.

The first parameter evaluated was the femtosecond laser phase time. This time is exclusive to the FLACS. In our experience, it has proven to be a highly reproducible time. It starts with the docking of the laser and ends with the release of suction at the end of the procedure. During this phase, the femtolaser performs in succession the anterior capsulotomy, the nucleus fragmentation, and the creation of corneal incisions. The mean time was 121.7 ± 27.3 s with minimal fluctuations in duration.

In all procedures, the integrity of the bulb was respected, maintaining a closed anterior chamber until the opening of the incisions by the surgeon. Few cases reported a partial loss of optimal mydriasis following the use of the femtosecond laser. As reported before, there was a single case of ineffective treatment out of 66 cases treated, with incomplete laser capsulotomy and nuclear fragmentation, which forced the surgeon to proceed with traditional surgery. This case was completed without any intra-operative complication, but the patient was excluded from statistical analysis.

The mean opening time of the corneal tunnel (about 2.1 mm) and the service incisions (about 1 mm) was 60.5 ± 20.4 s in the phacoemulsification standard procedure, while it was 48.8 ± 17.4 s in the femtosecond laser-assisted surgeries, with a statistically significant difference of 11.7± 16.5 s (19.3%, *p* = 0.04) in favor of the FLACS groups.

In the FLACS group, after the injection of viscoelastic (Duovisc^®^) in the anterior chamber, the surgeon manually lifted the anterior capsulotomy previously created by the laser. Among the 66 cases treated with the femtolaser, in 3 cases there was the presence of small ridges of the anterior capsulotomy, which didn’t affect the integrity of the procedure.

The creation of continuous curvilinear capsulorhexis was performed in PCS with Caporossi’s coaxial clamp for capsulorhexis.

The mean time of capsulorhexis was 39.6 ± 12.9 s in the PCS, while the mean time of the capsulotomy opening in FLACS was 7.0 ± 5.2 s. The mean difference between the two groups was 32.5 ± 6.9 s, which is a decrease of 83% of the time needed in FLACS compared with PCS (*p* < 0.0001).

The mean time of phacoemulsification was 180.1 ± 45.6 s in the PCS and 163.0 ± 38.2 s in the FLACS, with a mean difference of 16.6 ± 15.1 s (9.2%), which wasn’t statistically significant (*p* = 0.12). Nevertheless, the phacoemulsification time turned out to be much more reproducible in FLACS and more variable in the PCS.

The mean aspiration time of the residual cortical was 66.3 ± 27.5 s in the PCS and 91.5 ± 35.7 s in FLACS. This results in an additional 24.8 ± 29.6 s for the FLACS compared to the PCS, with a difference of 38% of the mean aspiration time (*p* = 0.02). No intraoperative complications occurred in this surgical step in either procedure.

Overall, the total surgical time of the cataract surgery was 742.3 ± 185.8 s in PCS and 985.1 ± 118.6 s in FLACS. The difference in the mean total operating duration between the two procedures is 242.8 ± 48.6 s, with a significant time reduction of 33% in PCS compared to FLACS (*p* = 0.03).

Nevertheless, the total operating time in FLACS included the time that the laser needed to perform the procedure (a mean time of 121.7 s as previously reported) and the time necessary to move the surgical bed from the laser workstation area to the surgical microscope area, as well as the preparation of a new sterile field. Moreover, FLACS allowed for a significantly higher reproducibility in surgical times with a significant reduction in time variance (34,521.6 s^2^ in PCS vs. 14,066.0 s^2^ in FLACS, *p* < 0.0001).

Finally, the mean CDE has been evaluated, and it was found to be 11.4 ± 5.6 in the PCS and 8.3 ± 3.4 in FLACS, with an excess of 27% of the energy dissipated during standard phacoemulsification in comparison to FLACS (*p* = 0.01).

Table 1 summarizes all surgical times in the two groups.

### Subgroups Analysis

A subgroup analysis compared the duration of the phacoemulsification phase to the cataract density (1+/2+ vs. 3+/4+ density).

Cataracts with densities 1+ and 2+ have been gathered in a first group, and those with densities of 3+ and 4+ in a second group.

In the FLACS group, we obtained a mean phacoemulsification phase time of 152.5 ± 25.4 s for 1+ and 2+ cataracts, compared with 176.0 ± 57.4 s for cataracts classified as 3+ and 4+. In the PCS group, the mean phacoemulsification phase time for cataracts classified as 1+ and 2+ was 124.8 ± 39.5 s, while for cataracts classified as 3+ and 4+, it was 281.3 ± 78.4 s. Those differences were significant between FLACS and PCS groups only in the 3+/4+ density group (*p* = 0.0001).

As expected, the higher density of the nucleus of the lens was associated with increased surgical times, but the rate of this increase differed between the two techniques: in the PCS group, 3+/4+ density was associated with a mean increase of 156.5 ± 24.8 s when compared to 1+/2+ density (an increase of 125.4% of phacoemulsification’s duration). On the other hand, in the FLACS group, the difference between 3+/4+ and 1+/2+ phacoemulsification times was only 23.5 ± 19.7 s, with an increase of 15.4% in surgical time.

Moreover, we found that in the FLACS, the duration of the phase of phacoemulsification is increased by 22.6% when compared to the PCS for cataracts with hardness 1+/2+. On the contrary, FLACS duration of phacoemulsification is greatly diminished for cataracts with hardness 3+/4+ (a mean reduction of 37.3%). This is in relation to the advantages obtained with the prior core-fragmentation phase operated by the femtosecond laser.

At last, the CDE parameter was significantly greater in the PCS group for both hardness 1+/2+ (9.3 ± 4.6) and also for hardness 3+/4+ (15.5 ± 6.4) when compared with the respective FLACS group (7.3 ± 2.8 in the 1+/2+ subgroup and 10.5 ± 4.8 in the 3+/4+ subgroup), with an increase of 22% (*p* = 0.02) and 32% (*p* = 0.01), respectively.

Subgroup analysis is summarized in Table 2.

## 4. Discussion

The current knowledge about femtosecond laser technology and its applications in cataract surgery has shown no significant differences from conventional phacoemulsification surgery concerning costs or clinical outcome, except in specific complex cataract scenarios. However, the industry is investing in this technology, and we could expect significant improvements in the near future [10,11]. Although it remains necessary to use the phacoemulsifier, the FLACS has shown benefits in terms of safety and reproducibility in systematic reviews [12].

In our study, we measured the time of each surgical phase. Several studies have observed that the main advantage obtained with the use of the femtosecond laser is the reduction of surgical time in the phases directly performed by the laser, such as the capsulotomy and the opening of the corneal tunnel and service incisions, as well as in the phase of phacoemulsification.

Although in our series we haven’t experienced any serious surgical complications, there has been a reduction in surgical time with the progression of the learning curve. The opening time of the service incisions has gradually reduced, and, with the increase in the number of cases treated, the need to use surgical blades to open the corneal incisions has drastically reduced. This seems to be the result of better control over the parameters of the energy used by the laser and a better interpretation of the positioning of corneal wounds. Every single femtosecond laser simply requires its own calibration. This is probably related to the characteristics of the machine and the environmental parameters of the operating room that houses it.

In all treated cases, the opening time of the laser capsulotomy was a repeatable result, and it was significantly lower than the manual curvilinear capsulorhexis performed in the control cases.

In our opinion, it seems that the main advantage of femtosecond cataract surgery is the reduction of the phacoemulsification time and the CDE parameter. Moreover, FLACS allows for higher accuracy and reproducibility of the surgical procedure, along with la ower reliance on surgeon experience.

In our experience, the reduction rate of the phase of phacoemulsification time was lower than in previous reports [13,14]. This is essentially due to the difference in the recording mode of the operating time, in our study, as previously reported, the duration of phacoemulsification was measured in its entirely, rather than just the time of actual ultrasonic pulse. The reduction of the duration clearly allows for advantages in terms of time, but especially the reduction of CDE means lower damage to the corneal endothelium and fewer endothelial cell losses [15], due to the lower energy dissipated during the phacoemulsification procedure. Various studies indicate that the ultrasonic energy released is also implicated in cystoid macular oedema pathogenesis; a reduction of CDE could then suggest a reduction in the incidence of this complication too [16].

The differences between the phacoemulsification time and the CDE become more evident in treating the hardest cataracts (3+/4+), in which we obtained a reduction of approximately 37% of the phacoemulsification time and 32% of the CDE. This reduction is due to the fragmentation of the cataract nucleus caused by the femtosecond laser, which facilitates the work of the phacoemulsifier in harder cataracts.

In our experience, separately considering the operating time of each phase revealed that only the suction time of the residual masses increased compared with the standard procedure. Residual masses in FLACS are more adherent to the capsular bag. Due to the presence of air bubbles in the capsular bag following the treatment with a femtosecond laser, the surgeon proceeds to a more delicate and less effective hydrodissection. It must be emphasized that the laser does not treat the posterior cortex of the lens for safety reasons, leaving it intact [17]. These two aspects, in our opinion, seem to explain why the aspiration of the residual mass requires more time in FLACS.

Although in three of the four operating phases analyzed the execution times of the procedures appear significantly reduced, the total duration of the intervention in FLACS is increased by about a third compared to the intervention in PCS. This is explained by considering that in FLACS there is additional time due to the execution of the laser procedure and the movement of the patient’s bed from the laser area to the surgical microscope, followed by the preparation of a new sterile field. The total mean duration of surgeries in our series was lower than in previous studies in literature [18].

Moreover, a recent clinical trial showed that, in comparison to FLACS, PCS showed a significantly higher cost-effectiveness ratio for every patient who had a successful treatment outcome. In light of this, despite having a more sophisticated technology, FLACS did not outperform phacoemulsification and, being more expensive, did not provide patients or healthcare systems with any significant advantages [19].

Although the small sample size remains the principal shortfall of our study, we believe our findings provide an insight into real-world surgical times of femtolaser assisted cataract surgery. Surely novel studies with a bigger cohort should be conducted in the future.

## Figures and Tables

**Table 1:** Surgical times in seconds in the two groups. FLACS = femtosecond assisted cataract surgery; PCS = standard phacoemulsification.

Time in Seconds	FLACS	PCS	*p*
Corneal tunnel and paracentesis preparation	48.8 ± 17.4	60.5 ± 20.4	0.04
Capsulorhexis/capsulotomy	7.0 ± 5.2	39.6 ± 12.9	<0.0001
Phacoemulsification	163.0 ± 38.2	180.1 ± 45.6	0.12
Residual cortical aspiration	91.5 ± 35.7	66.3 ± 27.5	0.02
Total surgery time	985.1 ± 118.6	742.3 ± 185.8	0.03

**Table 2:** Subgroup analysis based on cataract density (groups 1+/2+ vs. 3+/4+) using the two different surgical techniques. FLACS = femtosecond assisted cataract surgery; PCS = standard phacoemulsification; CDE = cumulative dissipated energy.

Parameters (±DS)	1+/2+ Density			3+/4+ Density		
	PCS	FLACS	*p*	PCS	FLACS	*p*
Phacoemulsification (s)	124.8 ± 39.5	152.5 ± 25.4	0.07	281.3 ± 78.4	176.0 ± 57.4	0.0001 *
CDE	9.3 ± 4.6	7.3 ± 2.8	0.02 *	15.5 ± 6.4	10.5 ± 4.8	0.01 *

* indicates statistically significant differences.

## Data Availability

Data can be obtained upon reasonable request to the corresponding author, M.M.C.
